# Corals Use Similar Immune Cells and Wound-Healing Processes as Those of Higher Organisms

**DOI:** 10.1371/journal.pone.0023992

**Published:** 2011-08-24

**Authors:** Caroline V. Palmer, Nikki G. Traylor-Knowles, Bette L. Willis, John C. Bythell

**Affiliations:** 1 ARC Centre of Excellence for Coral Reef Studies and School of Marine and Tropical Biology, James Cook University, Townsville, Queensland, Australia; 2 School of Biology, Newcastle University, Newcastle upon Tyne, United Kingdom; 3 Department of Biology, Boston University, Boston, Massachusetts, United States of America; Heriot-Watt University, United Kingdom

## Abstract

Sessile animals, like corals, frequently suffer physical injury from a variety of sources, thus wound-healing mechanisms that restore tissue integrity and prevent infection are vitally important for defence. Despite the ecological importance of reef-building corals, little is known about the cells and processes involved in wound healing in this group or in phylogenetically basal metazoans in general.

A histological investigation into wound healing of the scleractinian coral *Porites cylindrica* at 0 h, 6 h, 24 h and 48 h after injury revealed differences in cellular components between injured and healthy tissues. Cell counts of the obligate endosymbiont, *Symbiodinium,* and melanin volume fraction analysis revealed rapid declines in both *Symbiodinium* abundance and tissue cross-sectional area occupied by melanin-containing granular cells after injury. Four phases of wound healing were identified, which are similar to phases described for both vertebrates and invertebrates.

The four phases included (i) plug formation *via* the degranulation of melanin-containing granular cells; (ii) immune cell infiltration (inflammation); (iii) granular tissue formation (proliferation); and (iv) maturation. This study provides detailed documentation of the processes involved in scleractinian wound healing for the first time and further elucidates the roles of previously-described immune cells, such as fibroblasts. These results demonstrate the conservation of wound healing processes from anthozoans to humans.

## Introduction

Corals are frequently subjected to a variety of disturbances and events that cause wounding; further contributing to ongoing global declines in their health and abundance [1]. Wounds are defined as damage to or disruption of normal anatomical structure and function [2], and therefore include epithelial breaks, as well as extensive tissue damage and/or skeletal damage [3]. In corals, wounds may be caused by fish bites [4], algal abrasion and/or direct overgrowth [5], storm damage [6], [7], [8], and boring organisms [9]. However, histological studies of wound healing mechanisms in anthozoans are surprisingly few [10], [11], [12], [13] and most studies of wound healing in hard corals have focused on gross observations of regeneration rates under varying environmental conditions (e.g. [14], [15], [16], [17], [18]). Although histological aspects of wound healing in scleractinian corals have not been thoroughly investigated, they are likely to represent an important contribution to the understanding of coral immune responses, which in turn may lead to a better understanding of coral declines [19].

Wound healing is a vital process, during which specialised immune cells invade the wound site in a specific sequence [20] and seal the lesion to prevent the loss of fluids and infection by foreign organisms [21], [22], and to aid in regeneration of the tissue [14], [23]. In mammals, wound healing follows four phases that are broadly sequential, although overlapping [24], [25]. These same four phases have also been identified during wound healing within invertebrates [23], [26], [27], [28], [29], [30]. These studies demonstrate conservation of the key wound healing processes across several phyla, although there is little information on these processes in lower invertebrates (see [31]).

The first of the four wound healing phases, coagulation leading to clot formation, is similar among both invertebrates and vertebrates. Coagulation, whereby fluids such as blood or haemolymph become semisolid (soft clot) to seal the wound, begins immediately upon physical injury, in both vertebrates like humans [3] and invertebrates such as arthropods [21], [22]. Coagulation leads to the formation of a stable (hard) clot, where platelets aggregate, change shape and degranulate to form a plug [25], which is stabilised by fibrin molecules in mammals [3]. In insects, clot formation occurs by the degranulation of immune cells and the incorporation of cellular debris and extracellular matrix components into an extracellular aggregate [32], which may also be referred to as a “plug” [33].

After clot (or plug) formation, the second phase of wound healing is the infiltration of immune cells into tissues in the wound area, which is also referred to as the inflammation phase in vertebrates [3]. Infiltration of immune cells from tissue surrounding the wound site is triggered by immunity factors released by cells involved in clot formation, thereby initiating the inflammatory response [34] that comprises the second phase of wound healing [3]. During the inflammatory phase in both vertebrates [3] and invertebrates [29], [35], the recruited immune cells phagocytose microorganisms and cellular debris [25]. In the sea cucumber, *Holothuria polii*, for example, the second wound-healing phase has been identified by the infiltration of amoebocytes and pigment cells, and the presence of fibroblasts [35]. The presence of fibroblasts is also characteristic of this phase in mammals and signals the beginning of the third phase of wound healing, the proliferation phase [25].

The proliferation phase of wound healing, so named because of rapidly reproducing immune cells, primarily the fibroblasts, includes the development of granulation tissue and the process of re-epithelialisation in mammals [24]. Fibroblasts are characterised by discernable extended pseudopodia and an ability to control extra-cellular matrix production and collagen release [3], [36]. Proliferating fibroblasts are key to the perfusion of granulation tissue *via* the deposition of collagen, which is stimulated by various growth factors [20], [29]. Granulation tissue consists of multiple cell types and a basic extracellular matrix, which enables epithelial cells to migrate across it as part of the re-epithelialisation process [2], [3], [30]. During re-epithelialisation, epidermal cells proliferate and migrate from the wound edges and join. This occurs within a few hours in humans [3], after which tissue reorganisation begins underneath the newly formed epidermis [28]. In the insect *Drosophila*, proliferation of the epidermal cells does not occur during re-epithelialisation, rather, epidermal cells surrounding the lesion orientate towards the wound and extend cytoskeletal actin projections known as lamellipodia [33]. Similarly, orientation of epidermal cells at the lesion edge pointing towards the centre of the lesion has been documented in crayfish [37]. Additionally, Meszaros and Bigger (1999) noted an extension of epithelial tissue over the wound towards the exposed axial skeleton of the gorgonian sea fan *Plexaurella fusifera* within one day, although the mechanism was not described.

The fourth phase of wound healing is the maturation or remodelling phase, which is responsible for epithelium development and scar tissue formation [3], [24]. In mammals, this involves a reduction in fibroblast density *via* apoptosis [38], [39], which also serves to contract the wound, as well as extensive deposition of collagen [3]. For invertebrates, apoptosis of excess cells has been recorded [40] and remodelling involving collagen deposition, as part of regeneration, has been documented in the sea cucumber *H. glaberrima* [41] and leeches [42]. For anthozoans, reorganisation of new epithelium has been documented [12], and collagen production noted within lesions [10], however the potential presence of the aforementioned four phases of wound healing have not been investigated within hard corals. This study therefore aimed to investigate cellular mechanisms of wound healing histologically in the scleractinian coral *Porites cylindrica*. The results suggest that scleractinian wound healing occurs in phases similar to those described for higher invertebrates and vertebrates. Furthermore, several coral immune cells were identified and their roles in wound healing were documented.

## Results

### Symbiodinium density and melanin volume fraction

**Figure 1:**
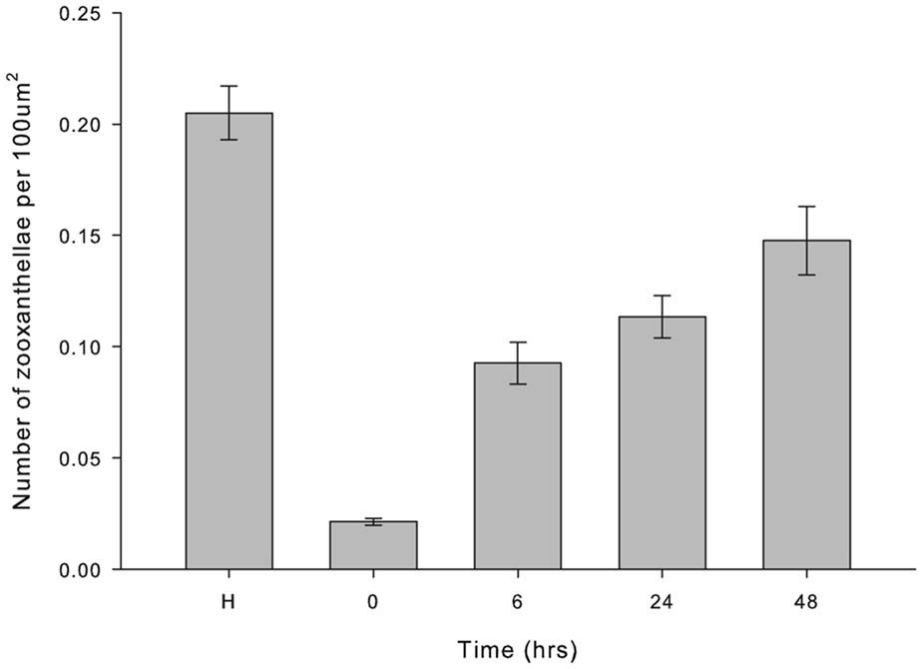
Mean zooxanthellae density (± SE) over time post-injury. H  =  healthy tissue, n =  27 (K-W *x* ^2^_(4)_  =  88.35, P<0.001).

Mean density of *Symbiodinium* cells was highest in healthy samples and varied significantly between healthy and injured samples (Figure 1; K-W *x* ^2^_(4)_  = 88.4, P<0.001). *Symbiodinium* density was lowest in freshly injured tissues (0 h), at 12.5% that of healthy tissue. Since sampling occurred adjacent to the area of full-thickness tissue removal (i.e. at the lesion edge), this reduction in *Symbiodinium* density (per unit gastrodermal tissue) cannot be directly accounted for by the removal of *Symbiodinium*-bearing tissue during wounding. The reduced *Symbiodinium* density therefore suggests a rapid release or expulsion of the endosymbionts after injury. After 1 h post-injury, there was an increase in *Symbiodinium* density over time, with a 4-fold increase between 1 h and 6 h post-injury and a further 1.5-fold increase between 6 h and 48 h post-injury. *Symbiodinium* density at 48 h post-injury was approximately 75% that of pre-injury cell density.

**Figure 2:**
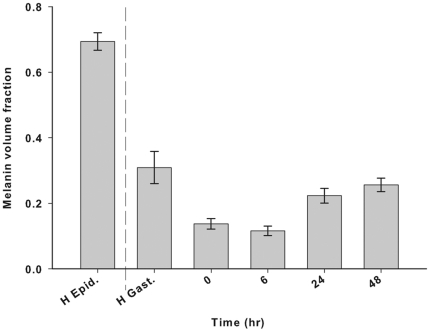
Mean melanin volume fraction (Vf; ± SE) over time post-injury (n =  27). H Epid  =  healthy coral epidermis. Melanin Vf compared among H. Gast. ( =  healthy gastrodermis) and gastrodermis within injured tissue (K-W *x* ^2^_(4)_  =  35.94, P<0.001).

The mean melanin volume fraction (Vf) differed significantly between gastrodermal tissues of healthy and injured samples over time post injury (Figure 2; K-W *x* ^2^_(4)_  = 35.9, P<0.001). Melanin Vf was highest in the epidermis of healthy samples, followed by the gastrodermis of healthy samples, where the Vf was approximately half that of the epidermis. Immediately after injury (0 h), melanin Vf in the injured gastrodermis decreased to approximately half that of the healthy gastrodermis Vf, and was approximately the same at 6 h post-injury. This suggests a rapid release of melanin from the tissues after injury. By 24 h, the melanin Vf had increased to approximately 80% of the Vf of the healthy gastrodermis and remained approximately the same at 48 h post-injury. This increase demonstrated a gradual replenishment of melanin to near that of healthy levels.

### Histopathology of wound healing

#### Healthy tissue

**Figure 3 pone-0023992-g003:**
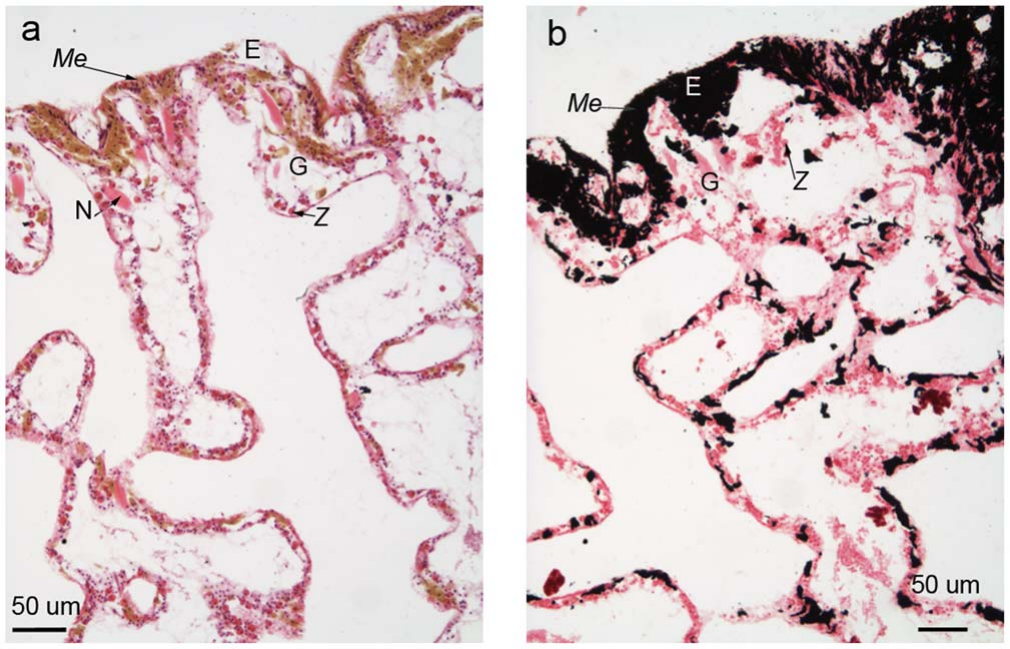
Healthy *Porites cylindrica* tissue sections stained with a) Haematoxylin and Young's Eosin-Erythrosin stain (H&E) and b) Fontana-Masson stain. E  =  epidermis, G  =  gastrodermis, *Me*  =  melanin and/or melanin-containing granular cell, Mes  =  mesentery, *Z*  =  zooxanthella (*Symbiodinium* cell*)* and N =  cnidae.

Histological sections of healthy tissues revealed intact epithelia that contained cells characteristic for each epithelium, for example, *Symbiodinium* cells within the gastrodermis (Figure 3). The epidermis was densely packed with melanin, visible within melanin-containing granular cells, although individual cells were difficult to discern due to their density. Melanin-containing granular cells were a characteristic deep golden brown colour in Haematoxylin and Young's Eosin-Erythrosin (H&E)-stained sections (Figure 3a) and black with the Fontana-Masson stain (Figure 3b). Cnidae of *Porites cylindrica* were large and dispersed primarily throughout the epithelia, although they were also observed occasionally within the mesenterial filaments.

#### Immediately post-injury (0 h)

**Figure 4 pone-0023992-g004:**
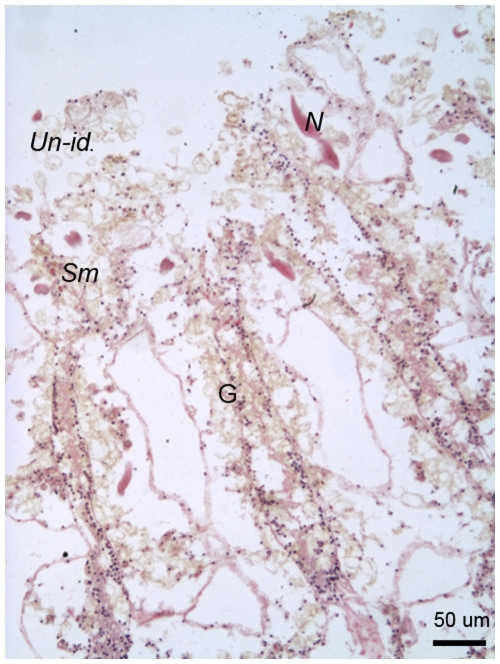
H&E stained free-body wall area of *Porites cylindrica* 0 h post-injury with surface body wall epidermis having been removed by the injury. G  =  gastrodermis, *Un-id.*  =  unidentified cells, *Sm*  =  smooth (hyaline) cell, *N = * cnida.

**Figure 5:**
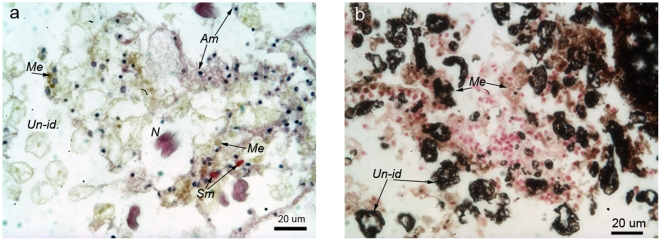
Disorganised tissues of *Porites cylindrica* immediately post-injury (0 h) stained with a) H&E and b) Fontana-Masson stain. *Un-id.*  =  unidentified cells, *Am*  =  granular amoebocytes, *Me*  =  melanin-containing granular cells, *Sm*  =  smooth (hyaline) cell, *N = * cnida.

Immediately post-injury, surface body wall epithelia were disrupted and cell layers could not be clearly differentiated. There was a notable lack of the melanin-containing granular cells characteristic of pre-injury tissues. Characteristic cell types present immediately post-injury included cnidae, which were relatively abundant, and *Symbiodinium*, which were in low density (Figure 4). Melanin granules were visible in H&E-stained sections (Figure 5a), and were associated with enlarged, round nuclei, discernable by purple to blue colouration under H&E. Light pink-stained (eosinophilic) cells with a granular appearance and dark oval nuclei were observed as putative amoebocytes. Hyaline (smooth) pink-stained cells were also present, as well as mucocytes and unidentified large cells, which appeared empty although with a slight brown colouration. The Fontana-Masson stained sections immediately post-injury indicated that melanin was associated with the empty-appearing unidentified cells, suggesting that they were degranulated melanin-containing granular cells (Figure 5b). Nuclei were stained red with the Fontana-Masson staining procedure due to counter-staining with Nuclear-fast red.

#### 6 hours post-injury

**Figure 6:**
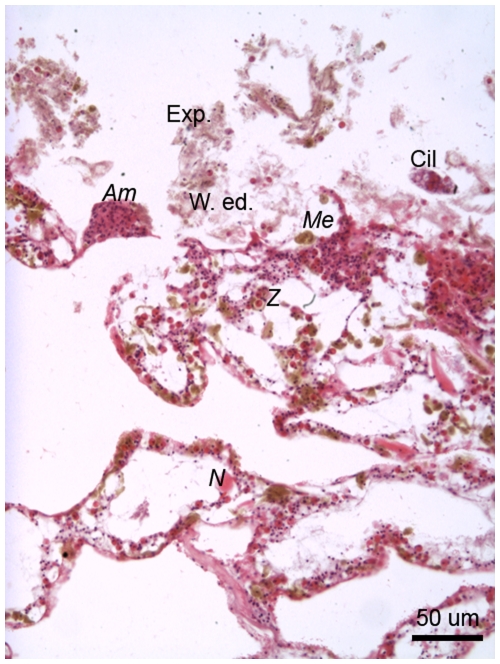
The edge of a lesion on *Porites cylindrica* 6 hours post-injury demonstrating amoebocyte aggregations, disrupted tissue and expelled, dead or dying tissue with microorganisms. Exp.  =  expelled, dead and dying tissue, W. ed.  =  wound edge, *Am*  =  granular amoebocytes, *Me*  =  melanin-containing granular amoebocytes, *Cil* =  ciliate, *N = * cnida.

Dark pink-stained (under H&E) highly granular cells observed at 6 h post-injury (Figure 6) were morphologically identical to the pale pink-stained eosinophilic amoebocytes that were observed at 0 h post-injury (Figure 5). This suggests a potential increase in the protein concentration of eosinophilic granular amoebocytes with time post-injury. Eosinophilic granular amoebocytes were in dense aggregations and established an epithelial front at the wound edge. These granular amoebocytes formed a boundary between the disrupted but live tissue and foreign organisms, such as ciliates, as well as the dead and expelled components that formed the cellular-debris plug (Figure 6).

**Figure 7:**
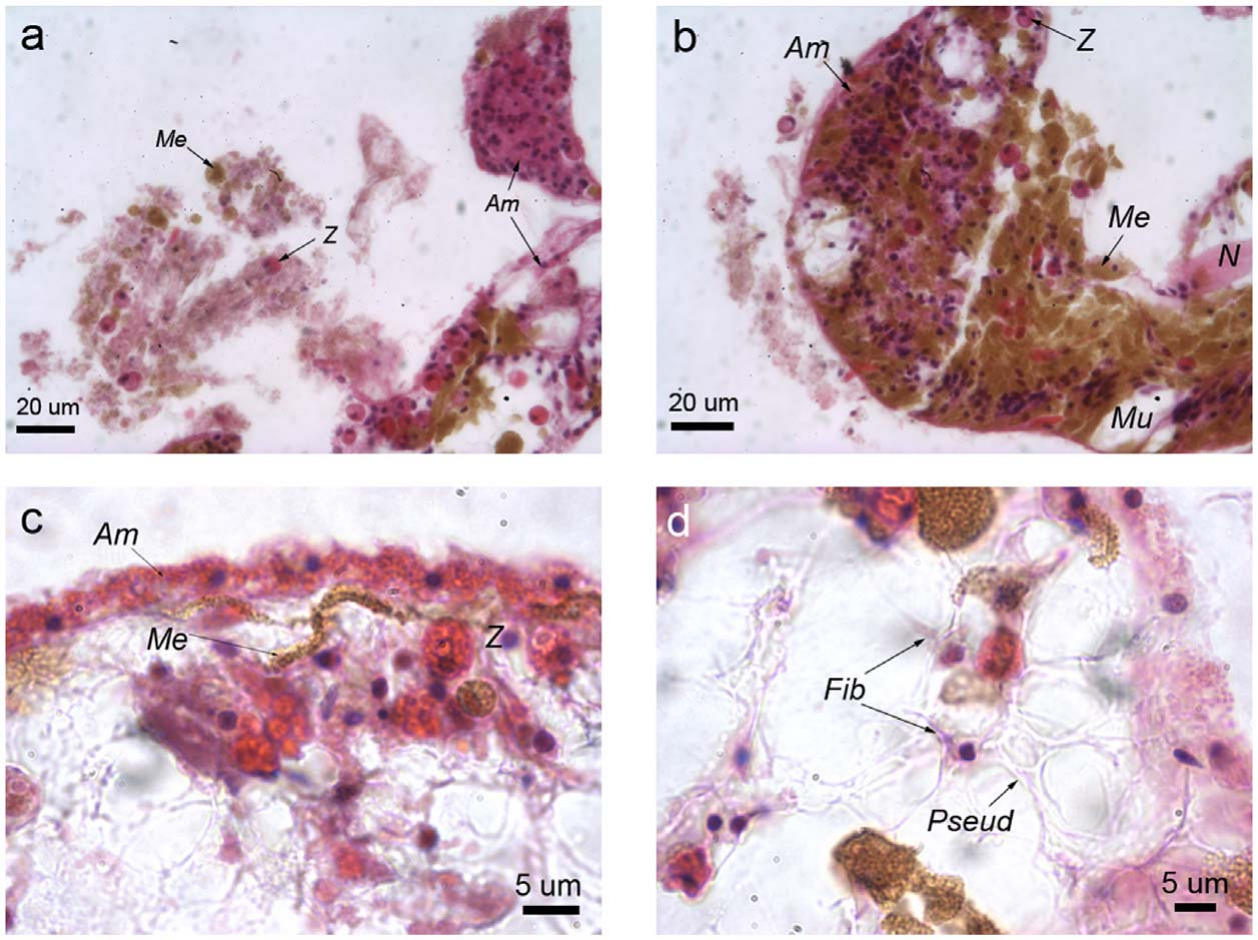
*Porites cylindrica* 6 hours post-injury with a) aggregating amoebocytes and expelled and/or disrupted tissue components, b) amoebocytes aggregating with melanin-containing-granular cells, c) granular amoebocytes forming a front, with melanin and *Symbiodinium* cells forming a second front immediately interior to the first one, and d) fibroblasts displaying characteristic pseudopodia potentially secreting connective collagen fibres. *Me*  =  melanin and/or melanin-containing granular cell, *N = * cnidae, *Mu*  =  mucocyte, *Am*  =  granular amoebocyte, *fib*  =  fibroblast and *Pseud*  = pseudopodia.

**Figure 8:**
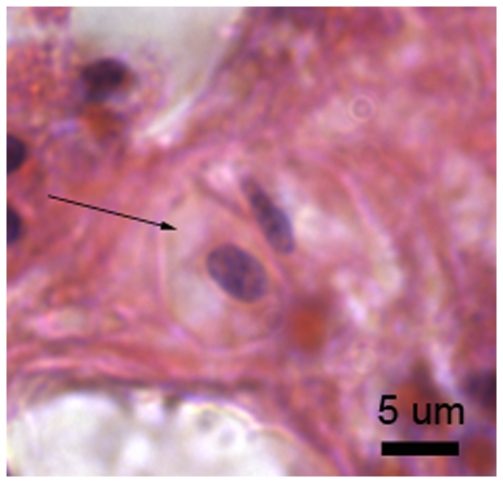
Agranular amoebocytes indicated with an arrow, present at 6 hours post-injury. Stained with H&E.

Expelled components that contributed to the plug included *Symbiodinium* cells, which were identifiable by their deep pink colouration (under H&E) and spherical shape, and melanin, potentially from degranulated melanin-containing granular cells (Figure 7a). Golden brown melanin-containing granular cells were densely aggregated with eosinophilic granular amoebocytes at the lesion edge (Figure 7b), where epithelial tissue layers remained indistinguishable, although *Symbiodinium* cells, smooth cells and cnidae were all present. A thin epithelial-like layer of eosinophilic granular amoebocytes formed along the lesion edge (Figure 7c), with melanin-containing granular cells forming a front directly interior to this layer. Also interior to the newly formed epithelial-like layer were agranular fibroblasts that had extensive pseudopodia, which connected the disorganised tissue components. The lack of staining with H&E indicated that the cytoplasmic content of the fibroblasts had a low protein concentration, potentially due to high levels of exocytosis of the synthesised collagen (Figure 7d). Simultaneously, dense aggregations of fibroblasts appeared to be laying down a collagen-like substance, as indicated by their light pink colouration under H&E. Agranular amoebocytes were also present, but in low densities and sporadically throughout the wound site (Figure 8).

#### 24 hours post-injury

**Figure 9:**
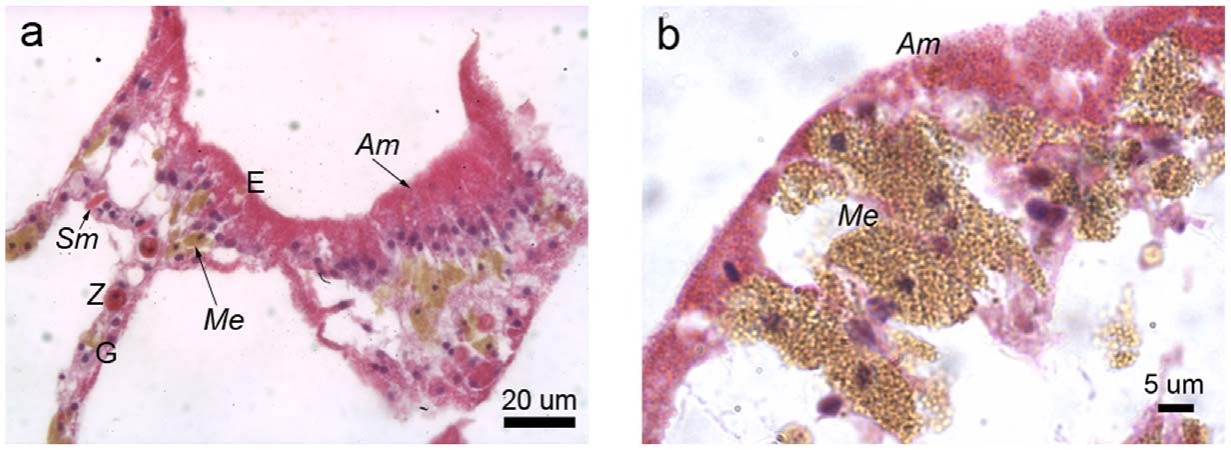
*Porites cylindrica* tissue 24 hours post-injury demonstrating a) granular amoebocytes aligning along the newly-forming epithelial layer, at the edge of the lesion b) granular amoebocytes being reinforced by melanin-containing granular cells. E  =  epidermis, G  =  gastrodermis, *Me*  =  melanin and/or melanin-containing granular cell, *Sm*  =  smooth (hyaline) cell, *Am*  =  granular amoebocyte.

At 24 h post-injury, granular amoebocytes were aggregated and aligned perpendicularly to the established epithelial-like front (Figure 9a). Epithelial layers, although not apparently separated by mesogloea, had begun to differentiate into gastrodermis and epidermis, with *Symbiodinium* cells located in the layer furthest away from the newly-forming epidermis (Figure 9a). Melanin-containing granular cells aggregated interior to the pink-stained (under H&E) granular amoebocytes (Figure 9b).

#### 48 hours post-injury

**Figure 10:**
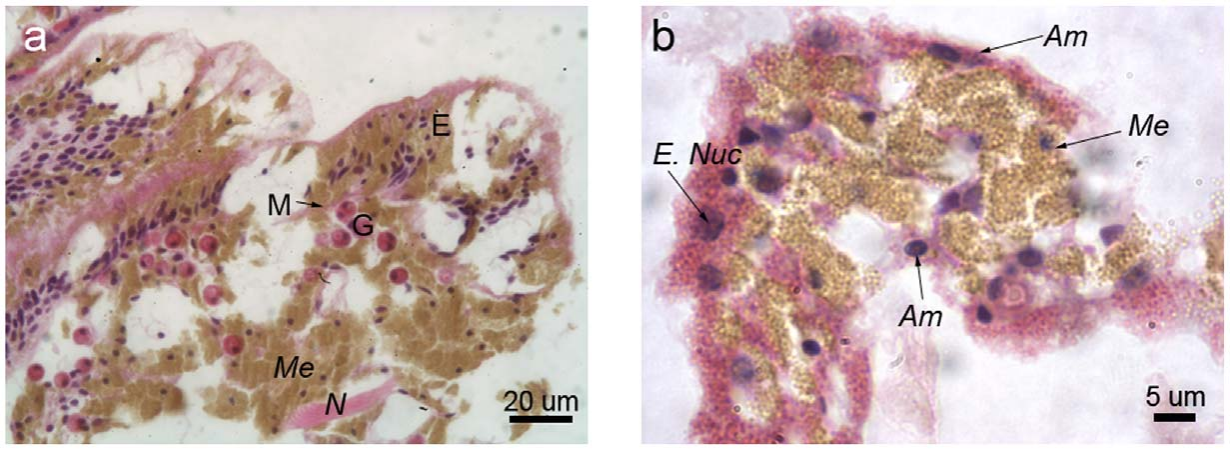
*Porites cylindrica* tissue 48 hours post-injury demonstrating a) a developing epithelial front and b) merging granular amoebocytes and melanin-containing granular cells. E  =  epidermis, M  =  mesogloea, G  =  gastrodermis, *Me*  =  melanin and/or melanin-containing granular cells, *N = * cnida, *E. nuc*  =  enlarged nucleus, *Am*  =  granular amoebocyte.

**Figure 11:**
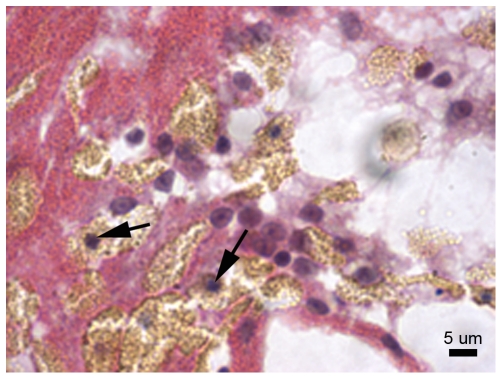
Potentially apoptotic cells as indicated by blebbing nuclei (arrow heads).

At 48 h post-injury, melanin-containing granular cells were in dense aggregations and the eosinophilic granular amoebocytes were in lower densities. The epithelial front was better developed than at 24 h post-injury, with epithelial layers distinguishable particularly at the lesion edge, and separated by a thin and developing mesogloea (Figure 10a). In less well-recovered areas, melanin-containing granular cells were observed behind the eosinophilic granular amoebocytes, as observed at 6 h and 24 h post-injury, although the distinction between the cell types was less clear (Figure 10b). Eosinophilic granular amoebocytes, previously observed as pink in colouration, contained golden-brown granules consistent with those of the melanin-containing granular cells (Figure 10b *Am*). This suggests that the eosinophilic granular amoebocytes may represent precursors of the melanin-containing granular cells. Additionally, several of these cells displayed morphological characteristics of apoptosis, such as blebbing and distinct dark blue (basophilic) aggregations within the cell (Figures 10b and 11), as previously described for anthozoans (e.g. [43], [44]).

#### Interpretation of the wound-healing process

**Figure 12:**
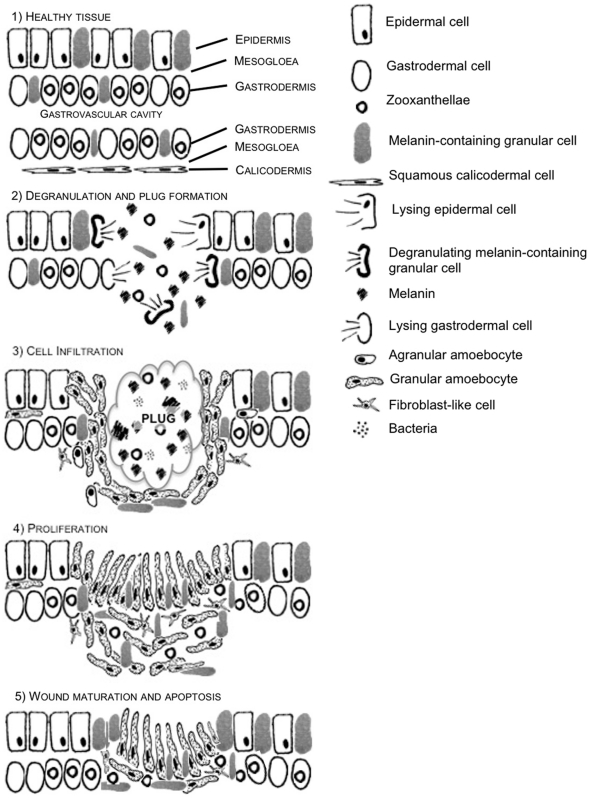
A series of schematic diagrams representing the wound healing process between 0 and 48 h in *P. cylindrica*. 1) Healthy coral tissue is composed of a surface body wall layer including the epidermis, a thin mesogloea and a gastrodermal layer. The epithelial layers are then mirrored on the other side of the gastrovascular cavity, to form the basal body wall, with a squamous epithelium forming the calicodermis. 2) Upon injury of the surface body wall epithelial layer, cell lysis and degranulation of melanin-containing granular cells starts to form a rudimentary clot, or plug. 3) Infiltration of granular amoebocytes, some potentially *via* the mesogloea, occurs at 6 h to form a cell barrier behind which melanin-containing granular cells aggregate, along with agranular amoebocytes and some fibroblasts. 4) At 24 h, infiltrated cells have begun to proliferate and the granular amoebocytes are reorientated and elongated to form an undifferentiated epidermis. 5) By 48 h, differentiation begins to occur and the epithelial layers begin to form from the lesion edge. The high abundance of amoebocytes and fibroblasts, potentially due to proliferation, is reduced by apoptosis.

A series of schematic diagrams (Figure 12), based on histological observations of wound healing in *Porites cylindrica*, depict the various stages that were observed. There were four discernable stages that relate to the established phases of wound healing identified in other animals (Figure 12, 2 to [Fig pone-0023992-g003] [Fig pone-0023992-g004] 5), from plug formation within the first hour post-injury, to wound maturation at 48 h post-injury.

## Discussion

Hard corals are subjected to physical damage from a variety of sources [4], [5], [6], [9] that injure tissues and cause open wounds. Injury is a frequent occurrence for some coral species, for example those that are heavily grazed or readily outcompeted, such as *Porites* spp. [4]. However, whole colony mortality is rarely a consequence of physical injury [7], [8]. Although the ability of coral colonies to regenerate lost tissue has been documented [14], [15], [16], [17], this study is the first to investigate and characterise in detail the cellular components and processes that enable effective wound healing in hard corals. Wound healing in *Porites cylindrica* in the 48 h following injury occurred in four main phases: i) degranulation and plug formation, ii) infiltration of immune cells, iii) cell proliferation, and finally iv) wound maturation *via* tissue layer differentiation and potentially apoptosis.

### Degranulation and plug formation: 0 hours post-injury

After a physical injury, the first phase of wound healing is clot formation [32]. In invertebrates such as butterflies and *Drosophila*, a soft clot or “plug” forms to seal the wound and establish haemostasis [32], [33], which is similar to clot formation in vertebrates [3]. This plug is formed of extracellular aggregates *via* degranulation of granular cells and the aggregation of cell debris [32]. Immediately after injury, there was a distinct lack of the characteristic golden-brown melanin-containing granular cells in *Porites cylindrica*, which coupled with the presence of extracellular melanin, demonstrates the degranulation of melanin-containing granular cells. The presence of both empty-appearing melanin-stained cells and exocytosed, extracellular melanin in histological sections suggests that these cells and cell debris contribute to the formation of a rudimentary clot or plug in *P. cylindrica*.

The melanin-containing granular cells observed in *P. cylindrica* wound healing appear similar to cells involved in wound healing in other organisms, for example, melanocytes described for mammals [45], [46] and melanophores described for frogs [47] and invertebrates, such as the fiddler crab [48]. Invertebrate granular cells, which are sometimes referred to as granulocytes, are extensively documented in literature on immunity [30], [49], [50], [51], [52], where they are reported to contain many active products involved in immunity, including phenoloxidase, melanin, peroxidase and lysozyme [21], [52], [53]. Degranulation of granular cells during wound healing [37], [52], [54], [55] releases antimicrobial and cytotoxic material, including melanin, which can kill foreign organisms. This mechanism is also documented for mammalian mast cells and granulocytes [45] and for crystal cells of insects [33], [56]. The degranulation of melanin-containing granular cells in *P. cylindrica* is consistent with soft clot or plug formation described for other invertebrates [27], [33].

Melanin-containing granular cell degranulation explains the immediate decrease in melanin volume fraction (Vf) in surrounding tissue and may explain decreases in *Symbiodinium* cell density. The local and immediate cytotoxicity induced as a result of melanin-containing granular cell degranulation is potentially responsible for the loss of *Symbiodinium*. The measured decrease in *Symbiodinium* cell density during wound healing in this study is contrary to results found in a study of gorgonian wound healing [12], in which symbiont densities were observed to increase. Furthermore, although wound-healing observations of *Plexaurella fusifera* began immediately after injury, there is no documentation of plug formation, only of disorganised tissue [12]. Similarly, plug formation or degranulation is not explicitly described for the anemone *Calliactis parasitica*, potentially because observations begin at 1 h post-injury [10]. However, Young (1974) notes the presence of a layer of cell debris and mesogloeal fibres covering the wound, which is similar to that observed for *P. cylindrica* [10].

### Infiltration of immune cells and the onset of proliferation: 6 hours post-injury

After a soft clot has formed during wound healing, immune cells infiltrate the area of injury as part of an inflammation response to resist infection [3], [29]. In *P. cylindrica,* there were dense aggregations of eosinophilic (acidophilic) granular amoebocytes, indicated by their deep pink staining with H&E by 6 hours post-injury. These eosinophilic granular amoebocytes are similar to those documented within the mollusc *Mytilus edulis*, where they are described to have phagocytic activity, and demonstrate strong superoxide radical production and phenoloxidase activity [51]. Due to these immunity-related activities and their ability to proliferate, eosinophilic amoebocytes are reportedly superior pathogen-killers to other immune cells [51], [52]. Although not conclusively established in this study, proliferation of eosinophilic granular amoebocytes would explain their high density in injured tissue sections, particularly as dense aggregations are not routinely observed in coral tissue sections [57], [58].

Eosinophilic granular amoebocytes observed in this study appear similar to the amoebocytes described during wound healing of *P. fusifera* [12] and to those observed during fungal infections of *Gorgonia ventalina* [59]. Meszaros and Bigger (1999) pointed out the similarities of amoebocyte nuclei morphology to those of epidermal cells, suggesting that they may play an important role in re-epithelialisation [12]. Consistent with these observations by Meszaros and Bigger (1999) and similar to observations of wound healing in other invertebrates e.g. [29], eosinophilic granular amoebocytes rapidly formed an epithelial-like layer by joining and flattening (spreading) along the lesion edge. This epithelial-like layer appeared to be reinforced by melanin-containing granular cells and appeared to represent the first stages of re-epithelialisation [1]. Re-epithelialisation observed in this study 6 hours post-injury in *P. cylindrica* is consistent with results reported for other invertebrates, such as *Drosophila,* for which epidermal cells migrated across the lesion within 8 hours [33], however, re-epithelialisation reportedly took 48 hours in the earthworm *Eisenia foetida* [28].

In addition to granular cells, hyaline cells, or hyalinocytes are another key group of immune cells and are described for molluscs [51], [52], [60], crustaceans [61] and cnidarians [10]. Hyaline cells are identified by their agranular cytoplasm and are round when unactivated or before spreading [52], consistent with the agranular cells observed at 6 hours post-injury in *P. cylindrica*. These round agranular anthozoan cells are comparable in morphology to mollusc haemocytes that stained positively for fibronectin and demonstrated fibroblast activity [29]. In *Porites cylindrica,* round agranular cells were in low density and were similar in size and in nuclear morphology to eosinophilic granular amoebocytes, melanin-containing granular cells and fibroblasts. These morphological consistencies among different cell types suggest that they originate from a common stem cell type that is triggered to differentiate during the various phases of wound-healing, as described within other invertebrates, including crayfish [62].

Fibroblasts are characterised as collagen and lipid-containing cells with the ability to fold membranes into discernable pseudopodia [3], [36]. Fibroblasts observed in *P. cylindrica* were agranular and had extensive pseudopodia that connected the disorganised tissue components. The presence of fibroblasts is consistent with wound healing in vertebrates, such as humans [3] and rats [63], as well as in invertebrates including the leech [36]. The role of fibroblasts appears consistent across the Metazoa, including being instrumental in reforming the extracellular matrix *via* the secretion of collagen. Fibroblasts are observed to infiltrate wound sites soon after injury and to indicate the onset of the proliferation phase [3], [36]. Although collagen staining was not conducted in this study, the lack of fibroblast staining with H&E indicates a low protein content in the cytoplasm, potentially due to high collagen synthesis.

### Proliferation and epidermis thickening at 24 hours post-injury

At 24 hours post-injury, the melanin volume fraction (Vf) was higher than at 6 hours post-injury, indicating an increase in melanin, potentially to provide structural support [26]. The newly formed epidermal-like layer of eosinophilic granular cells was thickened by this time point, which was consistent with observations of *P. fusifera* [12]. This observed thickening may be partly due to proliferation, but also the reorientation of cells and reorganisation of their morphology, from flattened cells aligned parallel to the lesion edge and joined end to end at 6 hours post-injury, to cells aligned perpendicular to the lesion edge and joined side-by-side at 24 hours post-injury. The latter of these cell organisation descriptions is characteristic of columnar cells of healthy coral epidermis. The disorganised cell layer located interior to the new epithelium in *P. cylindrica*, may represent maturing granulation tissue [3], which has been documented to develop in the mollusc *Limax maximus* at 24 hours post-injury [29]. However, circulatory components that are usually characteristic of granulation tissue [3], [29] were absent from *P. cylindrica* as they are not a normal part of anthozoan anatomy.

### Wound maturation and apoptosis at 48 hours post-injury

Consistent with recovering tissue, *Symbiodinium* cell density was higher at 48 h than at all earlier time points following injury, and granulation tissue had started to differentiate into discernable epithelia. However, eosinophilic granular amoebocytes also contained melanin granules and were in various stages of melanisation, suggesting that these cells may represent different stages of cell differentiation, as observed in the Sydney rock oyster [52] and crayfish [62]. Melanisation of eosinophilic granular amoebocytes suggests that their highly proteinaceous content may include prophenoloxidase, the precursor to melanin-synthesis, thus facilitating their conversion into melanin-containing granular cells. Similarly, given the appearance of the cytoplasm and nuclei, the agranular cells may be inactive forms of the fibroblasts [62].

The nuclei of a number of the eosinophilic granular amoebocytes at 48 h post-injury contained clumped material, which appeared as dark blue dots under H&E stain, potentially indicating apoptosis [37]. Apoptosis, or programmed cell death, is a component of wound healing, which eliminates excess cells that potentially have harmful content, in a non-damaging process [38]. In the crayfish, apoptosis of immune cells occurred four days after injury and was demonstrated by clumped nuclear material, including chromatin [37], consistent with observations in the current study.

### Conclusion

This study details the cellular process of wound healing in a hard coral for the first time and identifies four overlapping phases that are broadly consistent with those of organisms across a broad taxonomic range. This suggests that wound-healing processes are conserved across the metazoans, including the Anthozoa. Furthermore, immune cells of *P. cylindrica* were characterised and their functions identified, or proposed, for the first time. These cells include melanin-containing granular cells, eosinophilic granular cells, agranular cells and fibroblasts. Common characteristics between some of these cells suggest that they may originate from a common stem cell. Further studies into longer-term mechanisms involved in wound healing and comparative inter-specific studies would help to explain the differences in regeneration rates among coral species.

## Materials and Methods

### Sample collection

Three large (>50 cm diameter) and visually healthy colonies of *Porites cylindrica* were located and tagged within Pioneer Bay at Orpheus Island, Great Barrier Reef (GBR), during May 2008. Injuries were created on four branches per colony between 1 and 2 cm from the branch tip. To create the injury, bone-cutters were used to cut through tissue and skeleton to a depth of approximately 2 mm around the circumference of each branch. Injuries were created on branches that were on equivalent areas of each colony. Branches were sampled for histology using bone-cutters to remove the top 3 to 4 cm of each branch, inclusive of the injury. Samples were taken of a healthy branch from each colony (n = 3) as a control, then of injuries of each colony (n =  3) at 0 h, 6 h, 24 h and 48 h after injury.

### Histology

Samples of *Porites cylindrica* were fixed in 4% paraformaldehyde-phosphate buffer solution, decalcified progressively in 3% - 10% formic acid and stored in 70% ethanol. Samples were processed overnight in an automated tissue processor and embedded in paraffin wax. Subsamples of branches were sectioned longitudinally at 5 µm to give cross-sections of polyps, including polyps in the areas of injury. Sections were alternately stained with Mayer's Haematoxylin and Young's Eosin-Erythrosin stain (H&E) and Fontana-Masson melanin stain. Using an Olympus DP12 dedicated camera head mounted on an Olympus high power microscope, six photographs were taken of the injured area, which manifested as U-shaped indentations into the sides of longitudinal sections of branches, as *P. cylindrica* tissue extended deeper within the skeleton than the lesion. This enabled two photos of each side and the base of the U-shaped lesion (n =  6 photos) to be taken of each sample (n =  3 colonies), for each stain at both 20 x and 60 x magnification. Similarly, six photographs were taken haphazardly of the free body wall surface epithelia of healthy tissue sections for each colony. Cells of interest were photographed at 100 x magnification.

The number of *Symbiodinium* cells (zooxanthellae) per unit cross-sectional area of gastrodermis was counted within three randomly selected areas of gastrodermal tissue from the two sides and base of each U-shaped lesion (n =  9 areas per lesion), for one sample from each of the three colonies (n =  27 cell counts), from each time point (0 h, 6 h, 24 h and 48 h after injury). Similarly, *Symbiodinium* cells were counted within nine haphazardly selected areas of gastrodermis from each healthy sample (n =  27 cell counts). The tissue area was calculated using imaging software (Image J) and the mean density of *Symbiodinium* cells was compared statistically between healthy and injured samples over time using Kruskal-Wallis non-parametric tests, as assumptions of normality were not met.

The mean proportion of melanin in the tissue layers (volume fraction; Vf) was determined from Fontana-Masson stained sections, as a percentage surface area of melanin in the epidermis and gastrodermis of healthy tissue, and in disrupted tissue of injured samples, which resembled gastrodermis. Vf was calculated using Image J software by converting images to greyscale, and creating a histogram. The proportion of pixels within the selected area that were dark brown or black, thus indicating melanin, was recorded. Similar to counts of *Symbiodinium* cells, measurements of melanin Vf were made from three randomly selected areas of each side and the base of each lesion (n =  9 per lesion) for each colony (Tn =  27 measurements). Equivalent measurements were taken of the melanin Vf within the epidermis and gastrodermis of the healthy samples. Mean Vf of melanin was compared among time points using a Kruskal-Wallis non-parametric test.
